# Microwave-Assisted Synthesis of Chalcopyrite/Silver Phosphate Composites with Enhanced Degradation of Rhodamine B under Photo-Fenton Process

**DOI:** 10.3390/nano10112300

**Published:** 2020-11-20

**Authors:** Shun-An Chang, Po-Yu Wen, Tsunghsueh Wu, Yang-Wei Lin

**Affiliations:** 1 Department of Chemistry, National Changhua University of Education, 1 Jin-De road, Changhua City 50007, Taiwan; jack5566yes@gmail.com (S.-A.C.); bba02123@gmail.com (P.-Y.W.); 2 Department of Chemistry, University of Wisconsin-Platteville, 1 University Plaza, Platteville, WI 53818-3099, USA

**Keywords:** CuFeS_2_/Ag_3_PO_4_, Fenton process, degradation, sunlight, environmental water samples

## Abstract

A new composite by coupling chalcopyrite (CuFeS_2_) with silver phosphate (Ag_3_PO_4_) (CuFeS_2_/Ag_3_PO_4_) was proposed by using a cyclic microwave heating method. The prepared composites were characterized by scanning and transmission electron microscopy and X-ray diffraction, Fourier-transform infrared, UV–Vis diffuse reflectance spectroscopy, and X-ray photoelectron spectroscopy. Under optimum conditions and 2.5 W irradiation (wavelength length > 420 nm, power density = 0.38 Wcm^−2^), 96% of rhodamine B (RhB) was degraded by CuFeS_2_/Ag_3_PO_4_ within a 1 min photo-Fenton reaction, better than the performance of Ag_3_PO_4_ (25% degradation within 10 min), CuFeS_2_ (87.7% degradation within 1 min), and mechanically mixed CuFeS_2_/Ag_3_PO_4_ catalyst. RhB degradation mainly depended on the amount of hydroxyl radicals generated from the Fenton reaction. The degradation mechanism of CuFeS_2_/Ag_3_PO_4_ from the photo-Fenton reaction was deduced using a free radical trapping experiment, the chemical reaction of coumarin, and photocurrent and luminescence response. The incorporation of CuFeS_2_ in Ag_3_PO_4_ enhanced the charge separation of Ag_3_PO_4_ and reduced Ag_3_PO_4_ photocorrosion as the photogenerated electrons on Ag_3_PO_4_ were transferred to regenerate Cu^2+^/Fe^3+^ ions produced from the Fenton reaction to Cu^+^/Fe^2+^ ions, thus simultaneously maintaining the CuFeS_2_ intact. This demonstrates the synergistic effect on material stability. However, hydroxyl radicals were produced by both the photogenerated holes of Ag_3_PO_4_ and the Fenton reaction of CuFeS_2_ as another synergistic effect in catalysis. Notably, the degradation performance and the reusability of CuFeS_2_/Ag_3_PO_4_ were promoted. The practical applications of this new material were demonstrated from the effective performance of CuFeS_2_/Ag_3_PO_4_ composites in degrading various dyestuffs (90–98.9% degradation within 10 min) and dyes in environmental water samples (tap water, river water, pond water, seawater, treated wastewater) through enhanced the Fenton reaction under sunlight irradiation.

## 1. Introduction

Recently, concerns have been raised worldwide toward the harm caused by residual organic pollutants in surface water and groundwater, threatening ecosystems and aquatic species [1,2,3]. Among the many sources of water pollution, wastewater from the printing and dyeing industry is a major concern. The decolorization and degradation of most chromophores in dyes are difficult because of their stable aromatic structures, leading to prolonged toxic effects and environmental hazards [4,5,6]. Furthermore, dyes can absorb sunlight and reduce water clarity, preventing photosynthesis in aquatic plants, decreasing dissolved oxygen in water, affecting microbial diversity, and disrupting the self-purification capacity of water [7]. The removal of these deleterious and hazardous pollutants from industrial wastewater has become an urgent environmental need in the world [8].

Many studies have evaluated the removal of organic dyes by using photocatalytic degradation, ideally using sunlight, with the vision of sustainable water treatment. TiO_2_ is the most widely used photocatalytic semiconductor because of its nontoxic and stable nature, with a relatively low cost and abundant production resulting from a mature manufacturing process [9,10]. However, its absorption and photocatalytic activity depends entirely on ultraviolet light, which restricts its application in large-scale wastewater treatment [11,12,13]. Studies have been attempting to identify efficient sunlight-responsive photocatalysts, and Ag_3_PO_4,_ with a quantum efficiency of >90%, is considered an excellent candidate that is sunlight responsive [14,15,16]. However, it experiences considerable photocorrosion during photolysis. In addition to the photocatalytic method, Cu/Fe-bearing solids such as chalcopyrite (CuFeS_2_) have been widely used as catalysts in advanced oxidation processes (AOPs) for wastewater treatment [17,18,19,20,21]. In general, AOPs are based on Fenton’s chemistry, which utilizes hydroxyl radicals produced from the Fenton reaction between H_2_O_2_ and Cu^+^/Fe^2+^ ions to degrade organic dyes (Equations (1)–(6)) [22,23]. However, the reusability of CuFeS_2_ is a challenge due to its dissolution during water treatment and the slow kinetics of Fe^2+^ regeneration:

CuFeS_2_(s) + 8H_2_O_2_ → Fe^3+^ + Cu^2+^ + 2SO_4_^2−^ + 8H_2_O + 2H^+^ (1)

CuFeS_2_(s) + 16Fe^3+^ + 8H_2_O → 17Fe^2+^ +Cu^2+^ + 2SO_4_^2−^ + 16H^+^ (2)

CuFeS_2_(s) + 4O_2_ → Fe^2+^ + Cu^2+^ + 2SO_4_^2−^ (3)

Fe^2+^ + H_2_O_2_ → Fe^3+^ + OH^−^ + OH· (4)

Cu^2+^ + H_2_O_2_ → Cu^+^ + O_2_^−^· + 2H^+^ (5)

Cu^+^ + H_2_O_2_ → Cu^2+^ + OH^−^ + OH· (6)

Fe^3+^ + H_2_O → Fe(OH)^2+^ + H^+^ (7)

Fe(OH)^2+^ + hν → Fe^2+^ + OH· (8)

To address CuFeS_2_ reusability, attempts have been made to regenerate the Fenton catalysts with UV and/or visible-light irradiation, known as the photo-Fenton process [19,24,25]. Under UV and/or visible-light irradiation, Fe^3+^ complexes are formed from the Fenton reaction (Equation (7)) to produce both Fe^2+^ and hydroxyl radicals (Equation (8)). The photogenerated Fe^2+^ ions can catalyze the Fenton reaction to form Fe^3+^, thus demonstrating the recyclability of Fenton catalysts (Equation (4)). For instance, Dotto et al. demonstrated that the prepared citrate–CuFeS_2_ materials possessed 90% catalytic efficiency for bisphenol A (BPA) degradation with a 15 min photo-Fenton process for its rapid generation of hydroxyl radicals and efficient H_2_O_2_ consumption [24]. Dotto et al. also reported that the catalytic efficiency and stability was sustained after four consecutive photoregeneration cycles. However, the preparation of the novel CuFeS_2_ samples requires a high power and expensive microwave reactor (1400 W, 200 °C, 7 min). In another simpler attempt in material preparation, Pastrana-Martinez et al. used the mineral of CuFeS_2_ mined from Jendouba, Tunisia, to catalyze tyrosol (TY) degradation by using a UV light-emitting diode (LED)-assisted photo-Fenton reaction [19]. Higher total organic carbon (TOC) conversions (85.0%) and lower iron leaching (0.89 mg·L^−1^) were attained when the purified CuFeS_2_ samples were used in the photo-Fenton-like process within 60 min (0.50 mM TY at stoichiometric H_2_O_2_ concentrations). However, UV light irradiation is not considered sustainable because it requires a high energy input. The limitations of the methods used in all these studies underline the need to improve the visible-light absorption ability and efficiently boost the degradation performance and stability of CuFeS_2_. To our knowledge, CuFeS_2_ coupled with other semiconductors has not been examined in the Fenton process under visible-light irradiation.

Here, we synthesized CuFeS_2_ coupled with Ag_3_PO_4_ (CuFeS_2_/Ag_3_PO_4_) through cyclic microwave heating to address the challenges in material preparation, the stability of materials, and degradation performance. The breakthrough in our synthesis is that CuFeS_2_/Ag_3_PO_4_ could be prepared using a domestic 336 W microwave oven within 12 min. Our previous report indicated that Ag_3_PO_4_ is an efficient photocatalyst responsive to visible light and sunlight, so incorporating Ag_3_PO_4_ into the CuFeS_2_ Fenton reaction system might greatly increase the visible-light absorption while improving the regeneration of Fe^2+^/Cu^+^ ions. Here, we used CuFeS_2_/Ag_3_PO_4_ composites for the degradation of organic dyes (rhodamine B (RhB), methyl red (MR), rhodamine 6G (R6G), fluorescein, and propidium iodide (PI)). We also proposed that the degradation mechanism of CuFeS_2_/Ag_3_PO_4_ and the reactive species and successfully demonstrated the regeneration of the CuFeS_2_ catalyst and the application of CuFeS_2_/Ag_3_PO_4_ in the treatment of environmental samples.

## 2. Materials and Methods

### 2.1. Preparation of Ag_3_PO_4_, CuFeS_2,_ and CuFeS_2_/Ag_3_PO_4_

All chemicals were purchased from Sigma Aldrich (St. Louis, MO, USA) and were of analytical grade and thus used without further purification. First, 0.212 g of AgNO_3_ was added to 20 mL of deionized water (18.2 MΩ·cm) with stirring. Then, a colorless Ag(NH_3_)_2_^+^ ion solution was produced by adding 6.2 mL of 14 M NH_3_ solution dropwise into the AgNO_3_ solution. After 30 min stirring, 3.5 mL of H_3_PO_4_ (14.6 M) was used to neutralize the mixed solution to pH 7.0, and the solution was stirred again for 30 min in the dark. The yellow precipitate was filtered and subsequently rinsed with copies of deionized water and anhydrous ethanol. Finally, the as-synthesized Ag_3_PO_4_ was dried at 55 °C for 12 h [26].

CuFeS_2_ was prepared using the cyclic microwave heating method [27]. First, 19.7 mg of CuCl, 25.35 mg of FeCl_3,_ and 48.46 mg of L-cysteine were added to 20 mL of deionized water with stirring for 15 min. Then, the mixed solution underwent 10 cycles of 36 s heating and a 36 s pause in a domestic microwave oven (power: 336 W). After the black precipitate was cooled to room temperature, it was rinsed with deionized water and anhydrous ethanol. Finally, the as-synthesized CuFeS_2_ was dried at 55 °C for 12 h. Cu_2_S and Fe_2_S_3_ were prepared following similar method without adding FeCl_3_ and CuCl precursor, respectively. The CuFeS_2_/Ag_3_PO_4_ with a molar ratio of 2.5:1 was prepared as followed: 20 mg of Ag_3_PO_4_, 19.7 mg of CuCl, 25.35 mg of FeCl_3,_ and 48.46 mg of L-cysteine were added to 20 mL of deionized water with stirring for 15 min. Then, the mixed solution underwent 10 cycles of 36 s heating and a 36 s pause in a domestic microwave oven (power: 336 W). After the black precipitate was cooled to room temperature, it was rinsed with deionized water and anhydrous ethanol. Finally, the prepared CuFeS_2_/Ag_3_PO_4_ was dried at 55 °C for 12 h and the weight of CuFeS_2_/Ag_3_PO_4_ was 41.7 mg. Thus, the weight percentage of CuFeS_2_ in CuFeS_2_/Ag_3_PO_4_ was 52%. In this condition, we estimated the weight of CuFeS_2_ and Ag_3_PO_4_ in the CuFeS_2_/Ag_3_PO_4_ was 21.7 mg and 20 mg, respectively. Therefore, the molar ratio of CuFeS_2_/Ag_3_PO_4_ was calculated to be 2.5:1 [27]. In addition, the preparation of CuFeS_2_/Ag_3_PO_4_ with different molar ratios (0.4:1 and 1:1) followed the same method and was prepared by decreasing the adding amounts of CuCl and FeCl_3_ precursors at 20 mg Ag_3_PO_4_.

### 2.2. Characterization of Ag_3_PO_4_, CuFeS_2,_ and CuFeS_2_/Ag_3_PO_4_

The morphological and compositional characteristics of all as-prepared samples were observed with scanning electron microscopy (SEM) on a HITACHI S-4300 (Hitachi, Tokyo, Japan) and transmission electron microscopy (TEM) on a 1200EX II (JEOL, Tokyo, Japan) equipped with a QUANTAX Annular XFlash QUAD FQ5060 (Bruker Nano, Berlin, Germany). The crystallographic texture of the samples was measured through powder X-ray diffraction (XRD) on SMART APEX II (Bruker AXS, Billerica, MA, USA) using Cu Kα radiation (λ = 1.5406 Å). Fourier-transform infrared (FT-IR) spectra were obtained using an Agilent Cary 600 FT-IR spectrometer (Agilent Technologies, Santa Clara, CA, USA). An Evolution 2000UV–Vis spectrophotometer (Thermo Fisher Scientific Inc., Madison, WI, USA) with integrating spheres and reflectance standard material BaSO_4_ was applied to obtain the UV–Vis diffuse reflectance spectroscopy (DRS). The binding energy of elements was determined through X-ray photoelectron spectroscopy (XPS) on a VG ESCA210 (VG Scientific, West Sussex, UK).

### 2.3. Degradation Procedure by Using Ag_3_PO_4_, CuFeS_2,_ and CuFeS_2_/Ag_3_PO_4_

RhB degradation was used to assess the degradation activity of the prepared samples. The photoreactor PCX-50C (Beijing Perfectlight Technology Co. Ltd., Beijing, China) was equipped with a low-power white LED irradiation (2.5 W, power density = 0.38 W·cm^−2^, wavelength > 420 nm). For the photo-Fenton reaction, 20 mg of the prepared catalyst samples was added into the RhB solution (20 ppm, 50 mL), and the solution was stirred in the dark for 30 min. Before 10 min, to turn the light on and add H_2_O_2_, we measured the absorbance to check the adsorption ability of the prepared samples. Subsequently, the LED lamp was turned on and 200 μL of H_2_O_2_ (35%) was added. After given time intervals, 1 mL of suspension was taken, quenched immediately by adding 0.1 mM NaN_3_ and filtered by a 0.22 μm syringe filter organic membrane to remove the catalyst sample. The concentration of RhB was measured using a Synergy H1 hybrid multimode microplate reader (BioTek Instruments, Winooski, VT, USA) at its characteristic absorption peak of 550 nm. Similar processes were performed for other dyestuffs (MR, R6G, fluorescein, and PI). The RhB degradation (20 ppm, 50 mL) for the photocatalytic and Fenton reactions was performed using the same protocols (20 mg catalysts) as mentioned above, but without the addition of H_2_O_2_ and LED light irradiation, respectively. After the experiment, TOC concentration was determined on an Elementar Acquray TOC analyzer (Elementar Analysensysteme GmbH, Langenselbold, Germany) to evaluate the extent of mineralization.

### 2.4. Evaluation of Charge Separation and Recombination Rate of Ag_3_PO_4_, CuFeS_2,_ and CuFeS_2_/Ag_3_PO_4_

The charge separation efficiency and recombination rate of electron–hole pairs for the prepared composites were evaluated according to our earlier reports [28,29,30]. Photocurrent was measured to evaluate the charge separation efficiency of Ag_3_PO_4_, CuFeS_2_, and CuFeS_2_/Ag_3_PO_4_ composites under constant white LED irradiation at 60 s intervals. To determine the recombination rate of electron–hole pairs, the photoluminescence (PL) spectra of samples were obtained using λ_ex_ = 250 nm with a Varian Cary Eclipse fluorescence spectrophotometer (Agilent Technologies, Santa Clara, CA, USA).

### 2.5. Free Radical Trapping Experiment of Ag_3_PO_4_, CuFeS_2,_ and CuFeS_2_/Ag_3_PO_4_

To investigate the active species generated during RhB degradation over Ag_3_PO_4_, CuFeS_2_, and CuFeS_2_/Ag_3_PO_4_, the trapping experiment was conducted using ethylenediaminetetraacetate (EDTA), tert-butanol (t-BuOH), and p-benzoquinone (BQ) (each 1 mM) as the capturing agent for holes, hydroxyl radicals, and oxygen radicals, respectively. The implemented trapping experimental procedure was identical to the steps mentioned in the degradation section except for the capturing agent being added at each run.

## 3. Results and Discussion

### 3.1. Morphology and Crystal Phase of Ag_3_PO_4_, CuFeS_2,_ and CuFeS_2_/Ag_3_PO_4_

**Figure 1:**
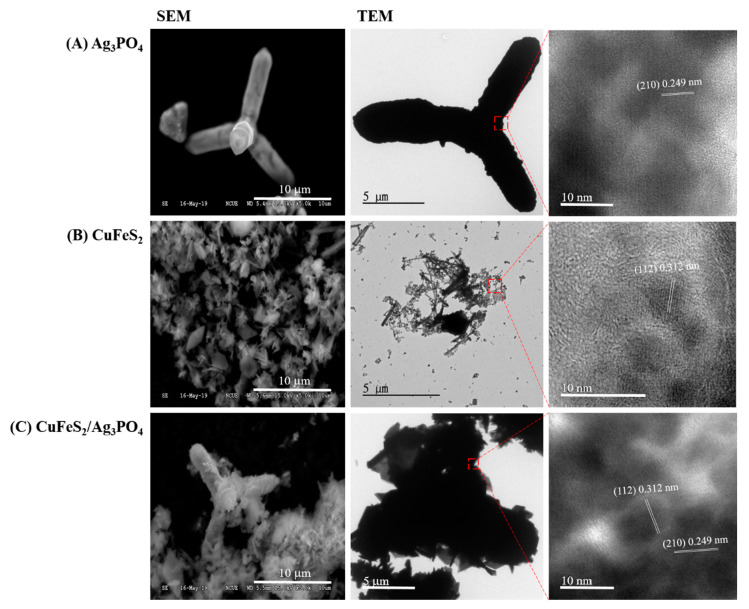
SEM and TEM images of (**A**) Ag_3_PO_4_; (**B**) CuFeS_2_; and (**C**) CuFeS_2_/Ag_3_PO_4_ composites.

**Figure 2:**
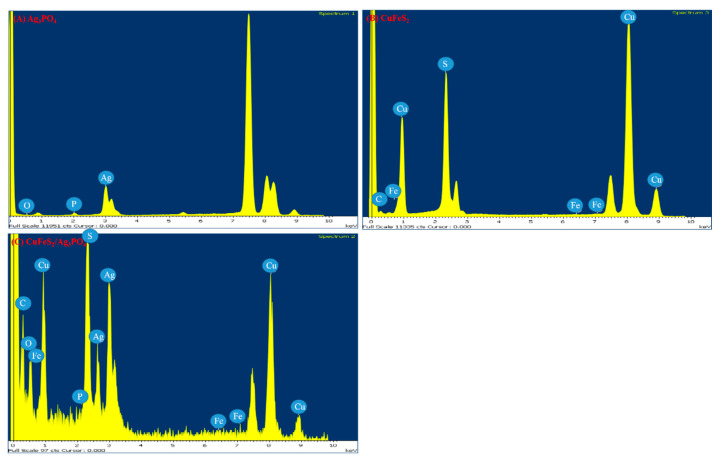
EDS spectra of (**A**) Ag_3_PO_4_; (**B**) CuFeS_2_; and (**C**) CuFeS_2_/Ag_3_PO_4_ composites.

The morphology of the prepared Ag_3_PO_4_, CuFeS_2,_ and CuFeS_2_/Ag_3_PO_4_ composites was analyzed through SEM and TEM (Figure 1). As shown in Figure 1, Ag_3_PO_4_ appears as tetrapod-like structures, with its four cylindrical arms being approximately 7–10 μm in length and with its average diameter being 2–3 μm. The CuFeS_2_ crystals appear irregular and sheet-like with sizes of 0.2–2 μm. As for the prepared Ag_3_PO_4_/CuFeS_2_ composites, CuFeS_2_ sheets were randomly deposited on the Ag_3_PO_4_ surface, and this deposition has no effect on the morphology and chemical composition of the Ag_3_PO_4_ particles. The corresponding magnified TEM image revealed clear lattice fringes with a d spacing of 0.249 and 0.312 nm, which are in good agreement with the (210) and (112) lattice planes of Ag_3_PO_4_ and CuFeS_2_, respectively. The energy dispersive spectrometer (EDS) spectra of the prepared Ag_3_PO_4_, CuFeS_2,_ and CuFeS_2_/Ag_3_PO_4_ composites (Figure 2) confirm the presence of Ag, P, O, Cu, Fe, and S elements in their crystals accordingly. In addition, we also found C element in the presence of CuFeS_2_ and CuFeS_2_/Ag_3_PO_4_ composites. This is because we used L-cysteine precursor in the preparation of CuFeS_2_ and CuFeS_2_/Ag_3_PO_4_ composites.

**Figure 3:**
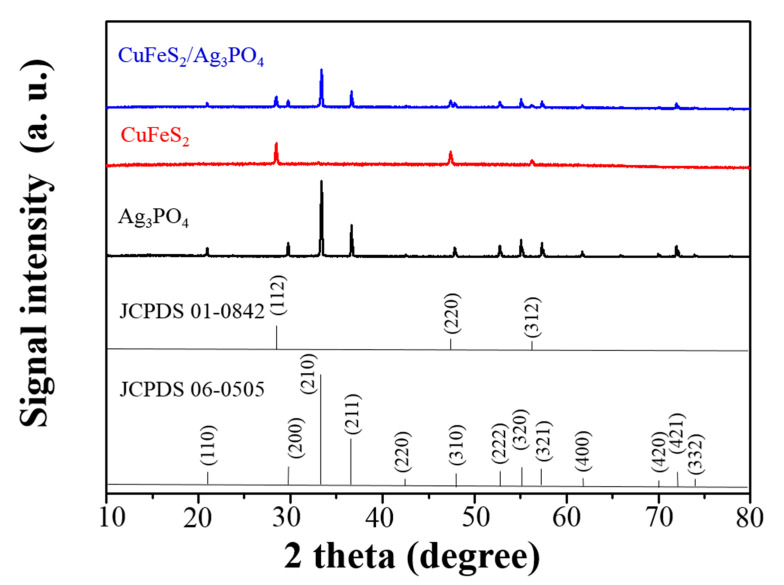
XRD patterns of different samples: Ag_3_PO_4_ (black); CuFeS_2_ (red); and CuFeS_2_/Ag_3_PO_4_ composites (blue).

The XRD spectra of the prepared Ag_3_PO_4_, CuFeS_2,_ and CuFeS_2_/Ag_3_PO_4_ composites are displayed in Figure 3. From the black curve of Ag_3_PO_4_, the diffraction peaks at 21.5°, 30.1°, 33.4°, 36.7°, 42.5°, 48.7°, 53.6°, 55.3°, 57.5°, 62.4°, 65.1°, 70.7°, 72.2°, 74.6°, and 78.2° were identified and assigned to the (110), (200), (210), (211), (220), (310), (222), (320), (321), (400), (330), (420), (421), (332), and (442) faces of the cubic Ag_3_PO_4_, respectively (JCPDS No. 06-0505) [26]. The XRD pattern of CuFeS_2_ is presented in the red curve of Figure 3, and the diffraction peaks at 28.9°, 46.4°, and 56.2° corresponded well to the (112), (220), and (312) faces of the tetragonal chalcopyrite CuFeS_2_, respectively (JCPDS No. 01-0842) [27]. As a confirmation of good composite quality, the diffraction patterns of the prepared CuFeS_2_/Ag_3_PO_4_ composites match with the patterns of Ag_3_PO_4_ and CuFeS_2_. According to Scherer’s equation, the average crystallite size of Ag_3_PO_4_ and CuFeS_2_ was 55.8 and 43.5 nm, respectively. In addition, they did not change in the CuFeS_2_/Ag_3_PO_4_ composites (Ag_3_PO_4_: 57.0 nm, CuFeS_2_: 46.5 nm).

### 3.2. Optical Property of Ag_3_PO_4_, CuFeS_2,_ and CuFeS_2_/Ag_3_PO_4_

**Figure 4:**
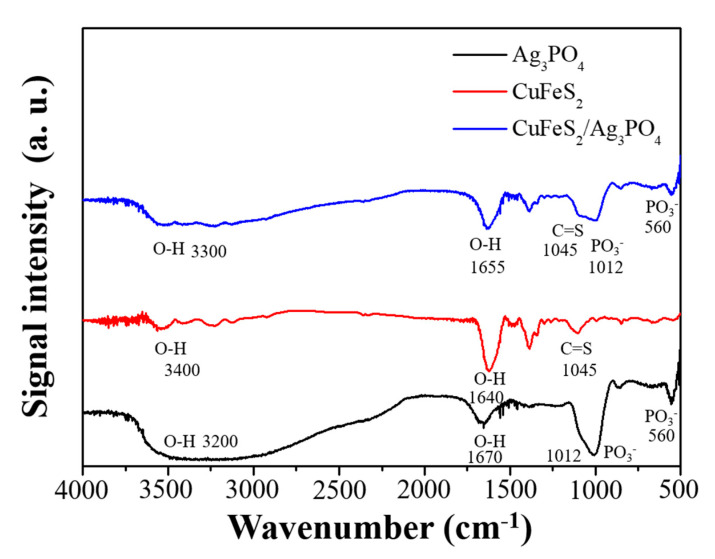
FT-IR spectra of different samples: Ag_3_PO_4_ (black); CuFeS_2_ (red); and CuFeS_2_/Ag_3_PO_4_ composites (blue).

The FT-IR spectra of the prepared Ag_3_PO_4_, CuFeS_2,_ and CuFeS_2_/Ag_3_PO_4_ composites are shown in Figure 4. The IR spectrum for Ag_3_PO_4_ shows four major peaks located at 560, 1012, 1670, and 3200 cm^−1^, which corresponded to the PO_4_^3−^ stretching and O–H bending vibration, respectively. The O–H bending in the prepared Ag_3_PO_4_ may be attributed from Ag_3_PO_4_ adsorbed water from the air. For CuFeS_2_, it shows three peaks located at 1045, 1640, and 3400 cm^−1^, which corresponded to the C=S stretching and O–H bending vibration, respectively. The O–H bending in the prepared CuFeS_2_ may be attributed from CuFeS_2_ adsorbed water from the air. The C=S stretching in the prepared CuFeS_2_ may be attributed from the L-cysteine precursor. In addition, the EDS spectra for CuFeS_2_ and CuFeS_2_/Ag_3_PO_4_ composites also confirmed the presence of C element. For CuFeS_2_/Ag_3_PO_4_ composites, the spectrum exhibited PO_4_^3−^, C=S, and O–H vibration peaks in the present on their crystals.

**Figure 5:**
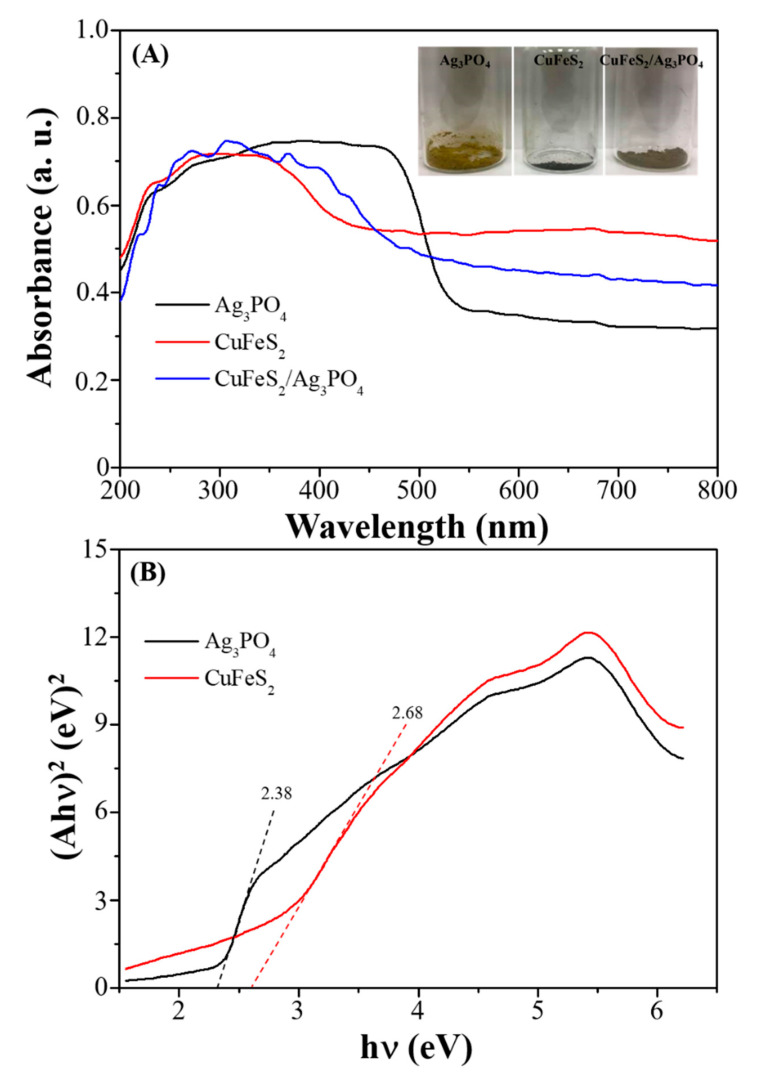
(**A**) UV–Vis diffuse reflectance spectroscopy (DRS) spectra and (**B**) Tauc’s plots of different samples: Ag_3_PO_4_ (black), CuFeS_2_ (red), and CuFeS_2_/Ag_3_PO_4_ composites (blue).

To study the optical absorption characteristics of the prepared Ag_3_PO_4_, CuFeS_2,_ and CuFeS_2_/Ag_3_PO_4_ composites, UV–Vis DRS was performed, as shown in Figure 5. The absorption band-edge for Ag_3_PO_4_, CuFeS_2_ and CuFeS_2_/Ag_3_PO_4_ is at approximately 525, 427, and 487 nm, respectively. The bandgap energy (Eg) of Ag_3_PO_4_ is theoretically estimated to be 2.38 eV using the equation αhν = A(hν − Eg)^n/2^ (n = 1 for Ag_3_PO_4_). Furthermore, the band-edge potentials for the conduction band (CB) and valence band (VB) can be calculated as E_VB_ = χ − E_H_ + 0.5 Eg and E_CB_ = E_VB_ − Eg, respectively; here, χ is the electronegativity of the constituent atom (5.96 eV for Ag_3_PO_4_) and E_H_ is the energy of the free electrons relative to the standard hydrogen reduction potential (approximately 4.5 eV). Thus, E_VB_ and E_CB_ of Ag_3_PO_4_ can be estimated to be 2.65 and 0.27 eV, respectively.

**Figure 6:**
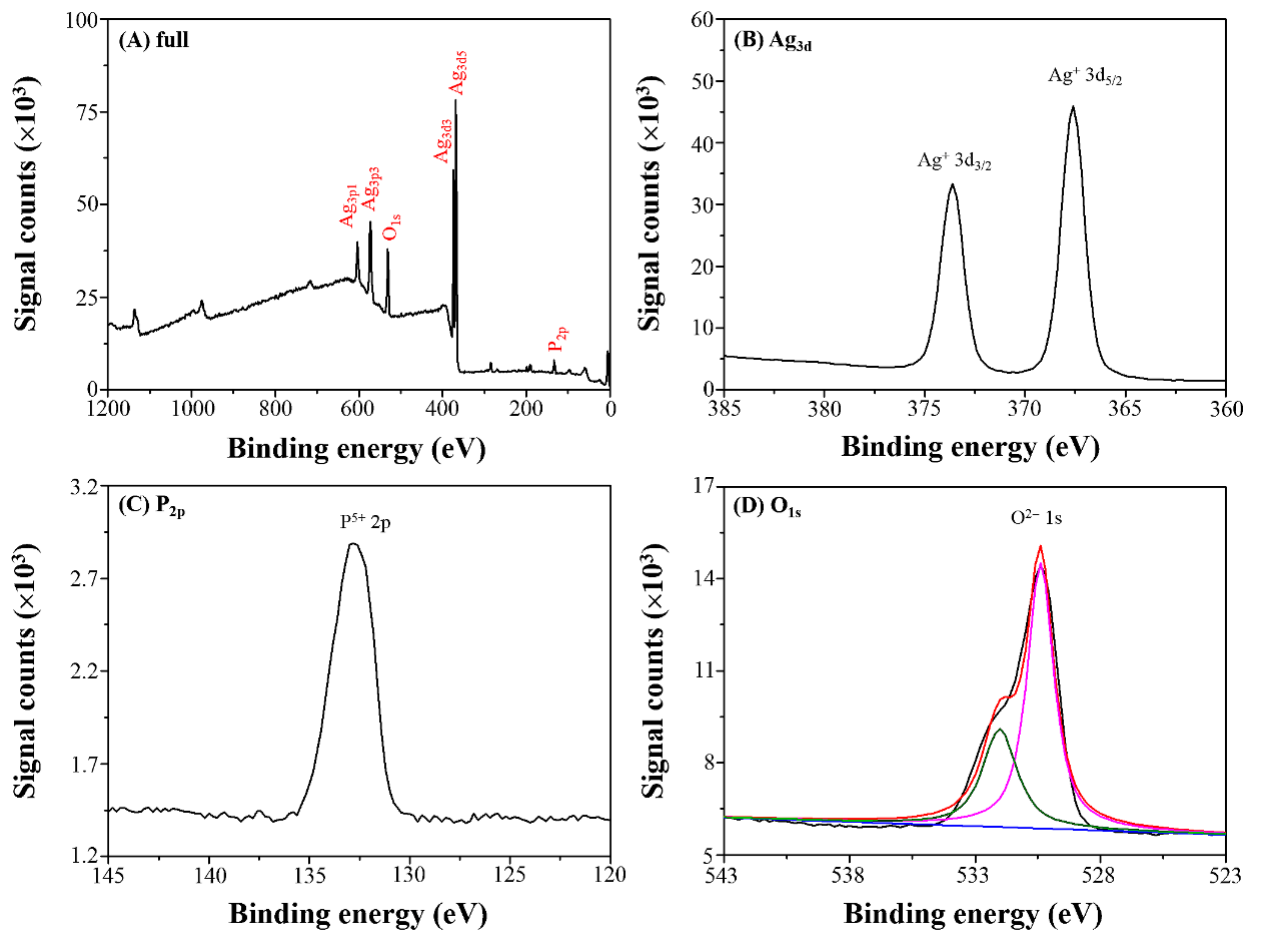
XPS spectra of Ag_3_PO_4_: (**A**) full scan; (**B**) Ag_3d_; (**C**) P_2p_; and (**D**) O_1s_. Deconvolution of XPS peak for O_1s_ element represented in different color line.

**Figure 7:**
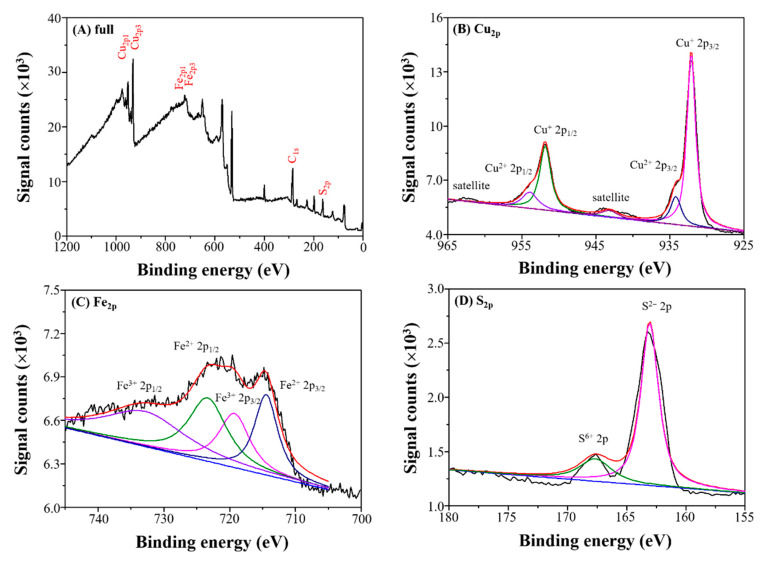
XPS spectra of CuFeS_2_: (**A**) full scan; (**B**) Cu_2p_; (**C**) Fe_2p_; and (**D**) S_2p_. Deconvolution of XPS peaks for Cu_2p_, Fe_2p_ and S_2p_ elements represented in different color line.

**Figure 8:**
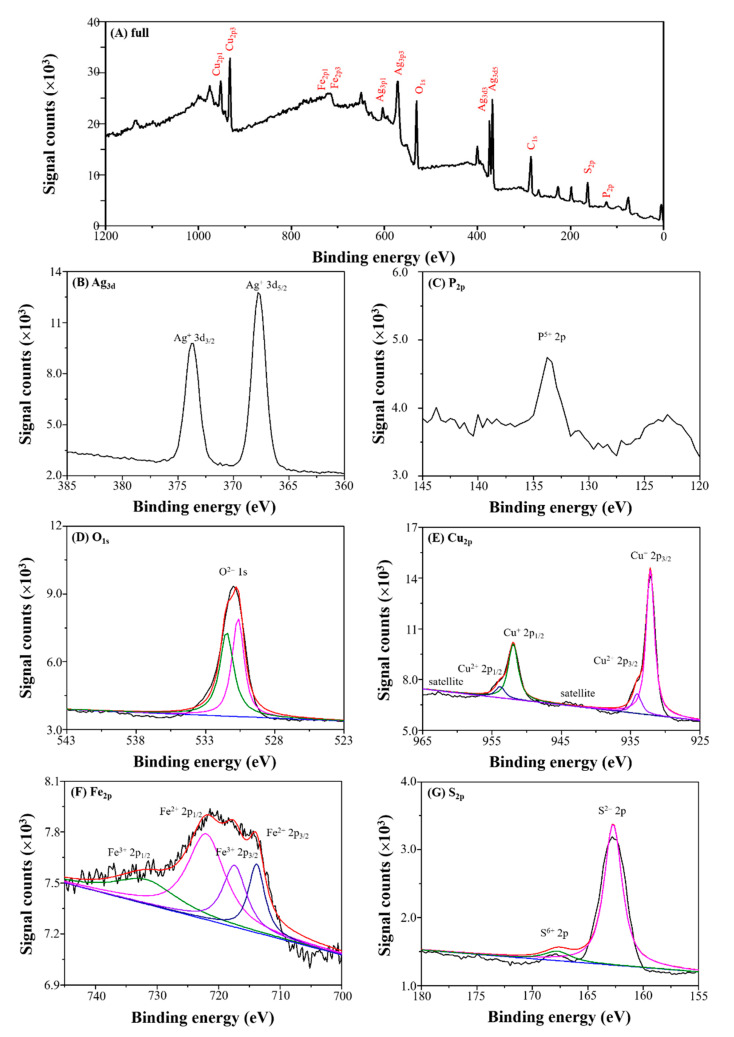
XPS spectra of CuFeS_2_/Ag_3_PO_4_: (**A**) full scan; (**B**) Ag_3d_; (**C**) P_2p_; (**D**) O_1s_; (**E**) Cu_2p;_ (**F**) Fe_2p_; and (**G**) S_2p_. Deconvolution of XPS peaks for O_1s_, Cu_2p_, Fe_2p_ and S_2p_ elements represented in different color line.

As another quality assurance method, the XPS analysis of the prepared Ag_3_PO_4_, CuFeS_2_, CuFeS_2_/Ag_3_PO_4_ composites (Figure 6, Figure 7 and Figure 8, full scan) revealed that the prepared composites contained their own elements: Ag, P, O, Cu, Fe, and S. High-resolution XPS revealed Ag_3d_, P_2p,_ O_1s_, Cu_2p_, Fe_2p_, and S_2p_ in CuFeS_2_/Ag_3_PO_4_ composites (Figure 8B–G, respectively). In Figure 8B, the binding energies are located at 367.8 and 373.3 eV, corresponding to Ag^+^ 3d_5/2_ and Ag^+^ 3d_3/2_, respectively. Furthermore, the peak at 132.9 eV correspond to P^5+^ 2p (Figure 8C). O 1s spectra can be deconvoluted into two component peaks of 531.8 and 530.7 eV (Figure 8D). The peak centered at 530.7 eV associated with the O_2_ in Ag_3_PO_4._ The other peak centered at 531.8 eV the presence of –OH group or a water molecule absorbed on the surface of the prepared composites. In Figure 8E, the peaks at 932.3, 934.2, 952.2, and 953.8 eV corresponded to Cu^+^ 2p_3/2_, Cu^2+^ 2p_3/2_, Cu^+^ 2p_1/2_, and Cu^2+^ 2p_1/2_, respectively. The peaks at 712.5, 718.2, 722.5, and 731.8 eV corresponded to Fe^2+^ 2p_3/2_, Fe^3+^ 2p_3/2_, Fe^2+^ 2p_1/2_ and Fe^3+^ 2p_1/2_, respectively (Figure 8F), whereas that at 162.5 and 167.8 eV corresponded to S^2−^ 2p and S^6+^ 2p, respectively (Figure 8G).

### 3.3. Degradation Performance of Ag_3_PO_4_, CuFeS_2,_ and CuFeS_2_/Ag_3_PO_4_

**Figure 9:**
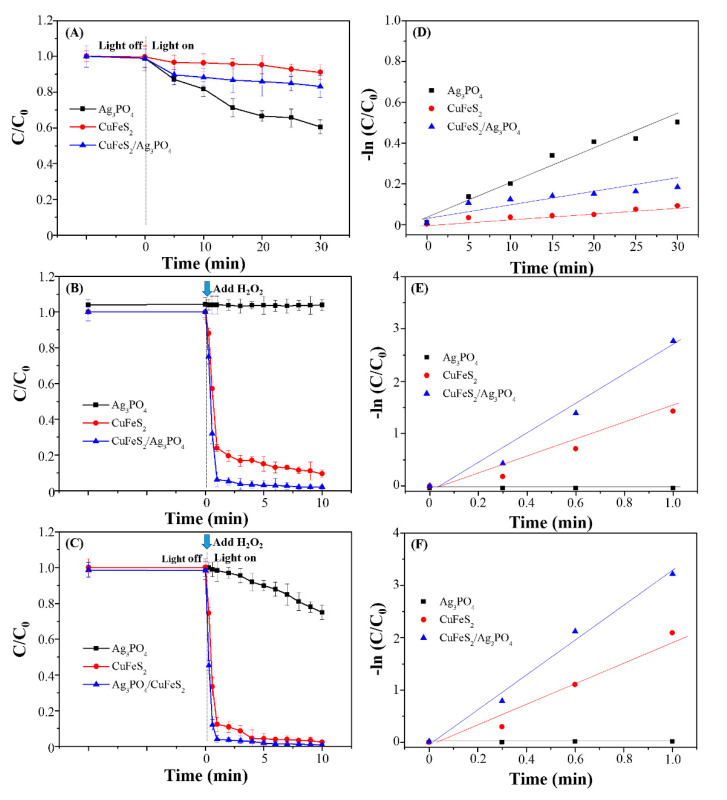
(**A**) Photocatalytic reaction; (**B**) fenton reaction; and (**C**) photo-Fenton reaction for RhB degradation by different samples: Ag_3_PO_4_ (black), CuFeS_2_ (red), and CuFeS_2_/Ag_3_PO_4_ composites (blue). The pseudo-first order linear transform of RhB degradation under (**D**) photocatalytic reaction, (**E**), Fenton reaction and (**F**) photo-Fenton reaction by different samples: Ag_3_PO_4_ (black), CuFeS_2_ (red), and CuFeS_2_/Ag_3_PO_4_ composites (blue).

**Figure 10:**
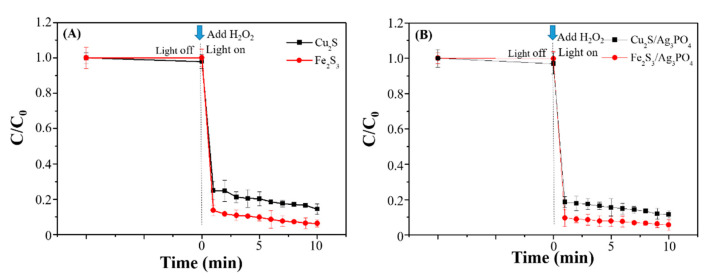
Photo-Fenton reaction for RhB degradation by different samples: (**A**) Cu_2_S (black) and Fe_2_S_3_ (red); and (**B**) Cu_2_S/Ag_3_PO_4_ (black) and Fe_2_S_3_/Ag_3_PO_4_ (red) composites.

To evaluate the degradation activity of the prepared Ag_3_PO_4_, CuFeS_2,_ and CuFeS_2_/Ag_3_PO_4_ composites, RhB was selected as the target pollutant. Figure 9 shows the concentration ratio (C/C_0_, where C_0_ and C represented the RhB concentration at the initial condition and at time t, respectively) of RhB by using Ag_3_PO_4_, CuFeS_2,_ and CuFeS_2_/Ag_3_PO_4_ composites under three different degradation conditions: photocatalytic, Fenton, and photo-Fenton. First, under 30 min LED light irradiation (Figure 9A), the degradation efficiencies of RhB in CuFeS_2_ and CuFeS_2_/Ag_3_PO_4_ are only 8.9% and 16.9%, respectively, whereas the efficiency reaches 39.5% in the presence of Ag_3_PO_4_. This suggests that the photocatalytic performance of Ag_3_PO_4_ is better than that of CuFeS_2_ and CuFeS_2_/Ag_3_PO_4_, attributable to the reactive radical formation from the effective photoinduced charge separation for RhB degradation. Nevertheless, these reactive radicals must be determined in a later experiment. CuFeS_2_/Ag_3_PO_4_ is responsive to visible light caused by its diluted amount of Ag_3_PO_4_, resulting in a weaker degradation performance than that of pure Ag_3_PO_4_. Second, the degradation efficiency of the prepared Ag_3_PO_4_, CuFeS_2,_ and CuFeS_2_/Ag_3_PO_4_ composites through the Fenton reaction in the dark was studied (Figure 9B). The RhB concentration barely changes in the presence of Ag_3_PO_4_, but the RhB degradation efficiency reaches 76.1% and 93.7% within 1 min in the presence of CuFeS_2_ and CuFeS_2_/Ag_3_PO_4,_ respectively. This is due to the production of hydroxyl radicals that degrade RhB from the oxidation of the Cu^+^/Fe^2+^ ions on the CuFeS_2_ to the formation of Cu^2+^/Fe^3+^ ions in the presence of H_2_O_2_. Finally, the degradation performance of the prepared Ag_3_PO_4_, CuFeS_2,_ and CuFeS_2_/Ag_3_PO_4_ composites was evaluated with the photo-Fenton reaction in the presence of H_2_O_2_, and the results are shown in Figure 9C. The degradation efficiency of Ag_3_PO_4_ reaches 25% with a 10 min photo-Fenton reaction, whereas that of CuFeS_2_ reaches 87.7% within 1 min. Notably, the degradation efficiency of CuFeS_2_/Ag_3_PO_4_ reaches 96% within 1 min. Moreover, the relative standard deviation of the degradation performance for three different batches of the prepared composites was less than 9%, indicating the high reproducibility of the preparation methods for the proposed composites. In addition, we also analyzed the degradation performances of Cu_2_S, Fe_2_S_3_, Cu_2_S/Ag_3_PO_4_ and Fe_2_S_3_/Ag_3_PO_4_ composites. As shown in Figure 10, the RhB degradation efficiency reaches 75.2%, 86.5%, 81.4% and 90.4% within 1 min in the presence of Cu_2_S, Fe_2_S_3_, Cu_2_S/Ag_3_PO_4_ and Fe_2_S_3_/Ag_3_PO_4,_ respectively. These results suggested that the addition of CuFeS_2_/Ag_3_PO_4_ promotes the production of hydroxyl radicals through the photo-Fenton reaction to degrade RhB at a higher efficiency than CuFeS_2_, Cu_2_S, Fe_2_S_3_ alone, indicating a synergistic effect between CuFeS_2_ and Ag_3_PO_4_. Figure 9D–F showed the corresponded pseudo-first order linear transform of RhB degradation under photocatalytic reaction, Fenton reaction and photo-Fenton reaction by Ag_3_PO_4_, CuFeS_2_, and CuFeS_2_/Ag_3_PO_4_ composites. The apparent rate constants for RhB degradation by Ag_3_PO_4_, CuFeS_2_, and CuFeS_2_/Ag_3_PO_4_ composites are listed in Table 1.

**Figure 11:**
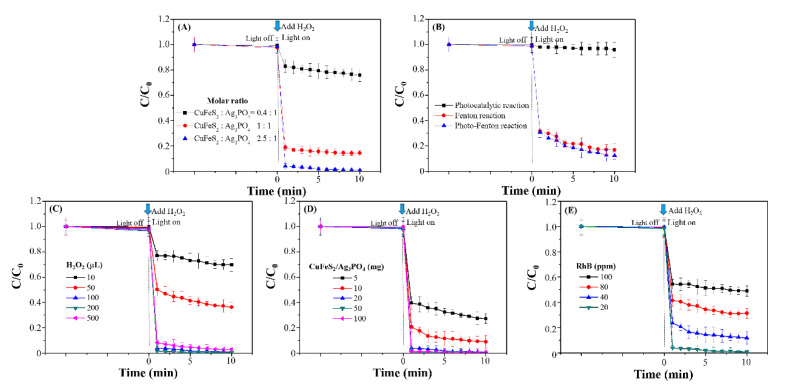
Degradation of RhB under different conditions: (**A**) different molar ratios of CuFeS_2_ to Ag_3_PO_4_; (**B**) different degradation procedures using the mechanical mixing of Ag_3_PO_4_ and CuFeS_2_ particles; (**C**) different amounts of H_2_O_2_ by CuFeS_2_/Ag_3_PO_4_ composites; (**D**) different amounts of CuFeS_2_/Ag_3_PO_4_ composites; and (**E**) different amounts of RhB.

To maximize the degradation performance of CuFeS_2_/Ag_3_PO_4_, the following factors were systematically studied: the molar ratio of CuFeS_2_ to Ag_3_PO_4_, the mechanical mixing of CuFeS_2_ and Ag_3_PO_4_ particles, and the added amounts of H_2_O_2_ and CuFeS_2_/Ag_3_PO_4_. Figure 11A presents the plots of the RhB concentration ratio (C/C_0_) against the reaction time for CuFeS_2_/Ag_3_PO_4_ prepared at different precursor molar ratios from CuFeS_2_ to Ag_3_PO_4_. The degradation efficiency can be visually related to the highest drop in the RhB concentration ratio in the plots during the reaction. The higher molar ratio of the precursors used to make CuFeS_2_/Ag_3_PO_4_ resulted in higher degradation efficiency as the hydroxyl radicals generated from the photo-Fenton reaction dominated the RhB degradation. Above the 2.5:1 ratio, the suspension of excessive black CuFeS_2_ particles in the CuFeS_2_/Ag_3_PO_4_ solution was observed. This raised a concern as the excessive CuFeS_2_ particles were not coupled with Ag_3_PO_4_ during the synthesis, and they cannot be regenerated during the photolysis. CuFeS_2_/Ag_3_PO_4_ with the molar ratio of 2.5:1 was therefore selected as the optimal condition for this study. The second experiment was performed by mechanically mixing both CuFeS_2_ and Ag_3_PO_4_ particles with the molar ratio of 2.5:1 to prove the synergistic effect within CuFeS_2_/Ag_3_PO_4_ in the enhanced degradation activity (Figure 11B). The degradation efficiency for the mechanically mixed sample with 10 min photocatalytic, Fenton, and photo-Fenton reactions is 4.2%, 83.5%, and 87.7%, respectively—all evidently lower than that for composites formed with cyclic microwave heating (Figure 9). These results strongly suggest that the coupling interaction between CuFeS_2_ and Ag_3_PO_4_ is critical for the significantly enhanced degradation activity of CuFeS_2_/Ag_3_PO_4_ composites. Then, because the amount of H_2_O_2_ is key for the photo-Fenton reaction, its content in the degradation process may influence the performance of CuFeS_2_/Ag_3_PO_4_ composites. As shown in Figure 11C, the degradation efficiency increased with an increase in the H_2_O_2_ amount up to 200 μL, above which the degradation efficiency decreased, probably because excess reactive radicals react with one another. Thus, we selected 200 μL of H_2_O_2_ as the optimum required amount of H_2_O_2_. Then, the degradation efficiency at different amounts of CuFeS_2_/Ag_3_PO_4_ composites was evaluated. As shown in Figure 11D, the degradation efficiency increased with an increase in the CuFeS_2_/Ag_3_PO_4_ amount of up to 20 mg, above which the degradation efficiency plateaued. On the basis of this result, 20 mg of CuFeS_2_/Ag_3_PO_4_ was used for further investigations. Finally, the degradation efficiency for a higher RhB concentration at 20 mg CuFeS_2_/Ag_3_PO_4_ was evaluated. As shown in Figure 11E, the degradation efficiency at a higher RhB concentration decreased within 10 min of photo-Fenton reaction. However, the degraded RhB amount was higher at a higher RhB concentration. The results also indicated that the maximum degraded amount of RhB at 20 mg CuFeS_2_/Ag_3_PO_4_ was 54.9 ppm.

**Figure 12:**
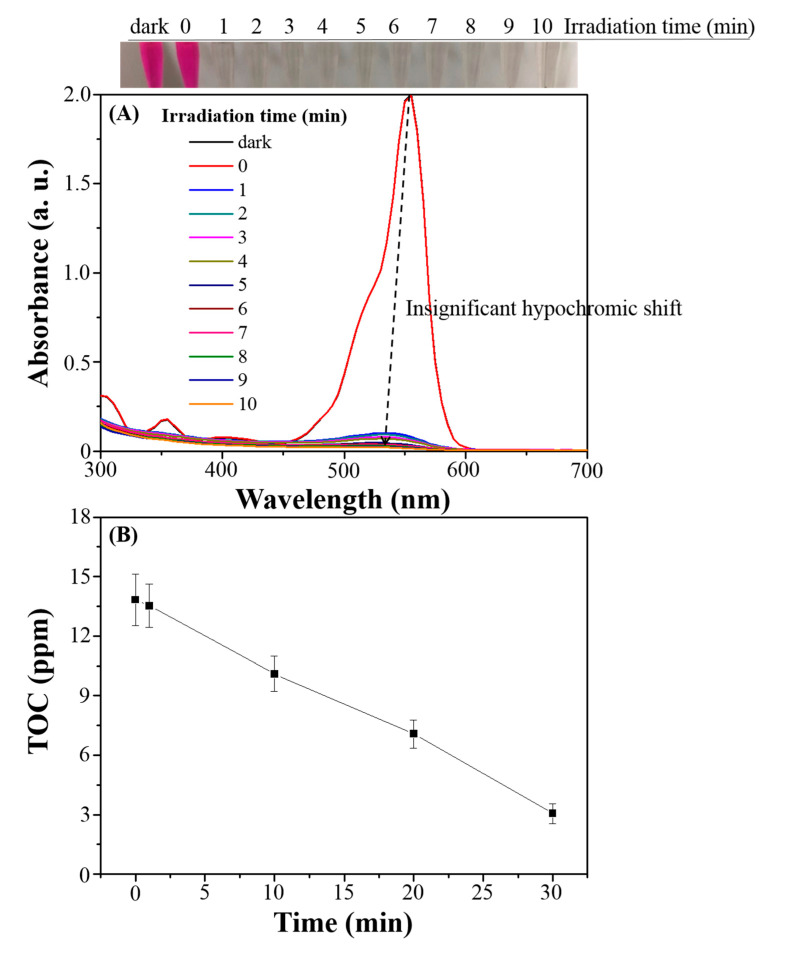
(**A**) Temporal evolution of UV–Vis spectra of RhB and (**B**) the corresponding total organic carbon (TOC) content of RhB degradation by the prepared CuFeS_2_/Ag_3_PO_4_ composites through the photo-Fenton reaction. Top image: photographs of the RhB solution under the photo-Fenton reaction at different irradiation time.

According to the literature, deethylation and chromophore cleavage are analogous competitive photodegradation reactions during the photocatalytic decomposition of organic pollutants. Based on the literature, the hypsochromic shifts (blue shift of the maximum absorption band) are attributed to the formation of a series of N-deethylated intermediates of RhB [31,32]. In this study, the absorption at 550 nm for RhB decreased with the increase in the reaction time and exhibited a slight hypochromic shift (dash line in Figure 12A). Therefore, a possible method to degrade RhB is through chromophore cleavage, which can be observed with an insignificant hypochromic shift in the UV–Vis spectra. Another approach to study organic compound degradation is to measure the TOC of the solution before and after the reaction. Figure 12B plots the temporal evolution of TOC content with RhB degradation by CuFeS_2_/Ag_3_PO_4_. Initially, the color vanished in the RhB solution with the photo-Fenton reaction for 1 min; the TOC content decreased to 13.5 ppm (decomposing only 2.17% of the original concentration (13.8 ppm)). The decomposition of RhB with CuFeS_2_/Ag_3_PO_4_ increased to as high as 78.3%, but only 3.0 ppm TOC remained in the solution with a 30 min photo-Fenton reaction. Evidence from both experimental approaches strongly supports RhB chromophore cleavage and CO_2_ production as the predominant pathways of degradation by CuFeS_2_/Ag_3_PO_4_.

### 3.4. Degradation Mechanism of Ag_3_PO_4_, CuFeS_2,_ and CuFeS_2_/Ag_3_PO_4_

**Figure 13:**
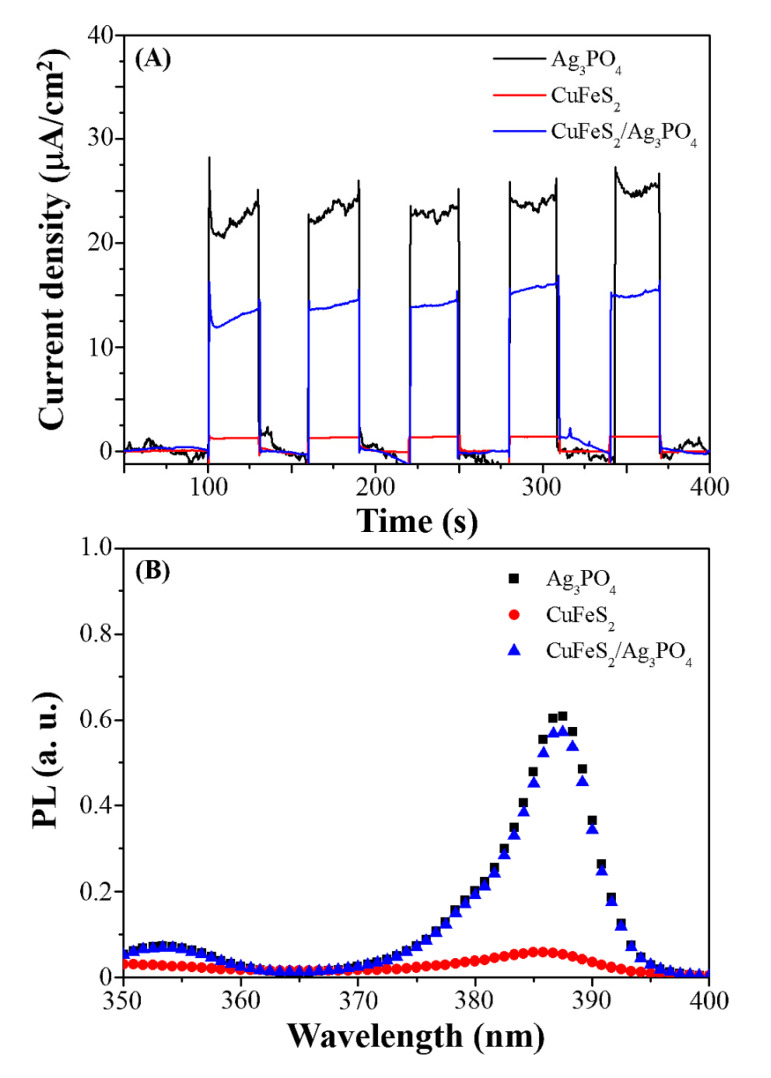
(**A**) Photocurrent density and (**B**) the photoluminescence (PL) spectra of different samples: Ag_3_PO_4_ (black), CuFeS_2_ (red), and CuFeS_2_/Ag_3_PO_4_ composites (blue).

In photodegradation, the rate and the stability of electron–hole pairs generated from the excitation source are critical factors to be considered in the development of ideal photocatalysts. In this study, the separation efficiency and recombination rate of the electron–hole pairs for the prepared Ag_3_PO_4_, CuFeS_2_, and CuFeS_2_/Ag_3_PO_4_ composites were investigated through photocatalytic degradation without any H_2_O_2_. Figure 13A shows the photocurrent density of Ag_3_PO_4_, CuFeS_2_, and CuFeS_2_/Ag_3_PO_4_ under LED light irradiation; all samples produced a sharp photocurrent peak on turning the light on and no peak as the light turned off, with photocurrent response during the on/off cycles. Under LED light irradiation, the current density decreased in the following order: Ag_3_PO_4_ > CuFeS_2_/Ag_3_PO_4_ > CuFeS_2_. This result is consistent with the photocatalytic degradation results shown in Figure 9A, indicating that the differences in the photodegradation activity was due to the differences in the optoelectrical properties of selected materials. In addition, the different noise levels and shape forms of the photocurrent density for different composites may be attributed to the different size and morphology of the composites deposited on the fluorine-doped tin oxide (FTO) substrates. As a further experiment, the intensity of PL emission for three materials was measured to quantify the recombination rate of the electron–hole pairs; a lower rate implies a lower luminescence emission intensity and higher photocatalytic activity. Figure 13B presents the PL emission spectra of the prepared Ag_3_PO_4_, CuFeS_2_, and CuFeS_2_/Ag_3_PO_4_ composites at λ_ex_ = 250 nm. The emission intensity of CuFeS_2_ was the weakest due to the lowest yield of electron–hole pairs but not due to a low recombination rate. The difference in the emission intensity between Ag_3_PO_4_ and CuFeS_2_/Ag_3_PO_4_ is nonsignificant, indicating comparable electron–hole pair recombination rates. This demonstrates the ability of CuFeS_2_/Ag_3_PO_4_ to generate electron–hole pairs with a low recombination rate, providing critical information to understand the mechanism discussed later.

**Figure 14:**
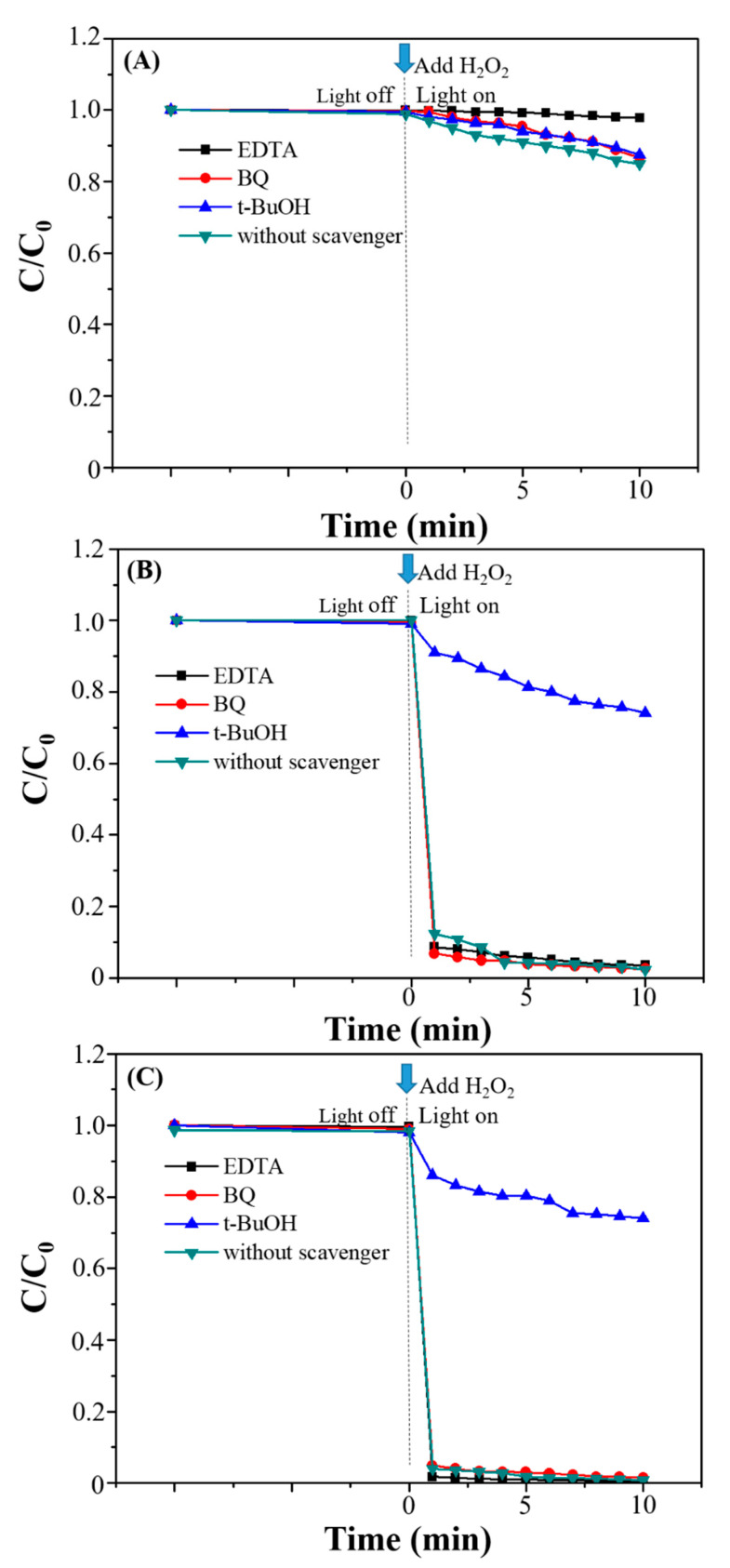
Free radical trapping experiment of (**A**) Ag_3_PO_4_, (**B**) CuFeS_2_, and (**C**) CuFeS_2_/Ag_3_PO_4_ composites. Ethylenediaminetetraacetate (EDTA), tert-butanol (t-BuOH), and p-benzoquinone (BQ) (each 1 mM) were scavengers for holes, oxygen radicals, and hydroxyl radicals, respectively.

As a key mechanistic study, the active species involved in the degradation reaction were identified systematically using the free radical trapping experiments (Figure 14). EDTA, BQ, and t-BuOH were used as holes, oxygen radicals, and hydroxyl radical scavengers, respectively. After adding EDTA to the reaction mixture, RhB degradation in Ag_3_PO_4_ samples was inhibited, indicating that holes are the major species involved in the photo-Fenton degradation (Figure 14A). In contrast to the Ag_3_PO_4_ sample, RhB degradation in CuFeS_2_ and CuFeS_2_/Ag_3_PO_4_ composites with the photo-Fenton reaction was inhibited by adding t-BuOH, indicating that hydroxyl radicals are the major active species (Figure 14B,C).

**Figure 15:**
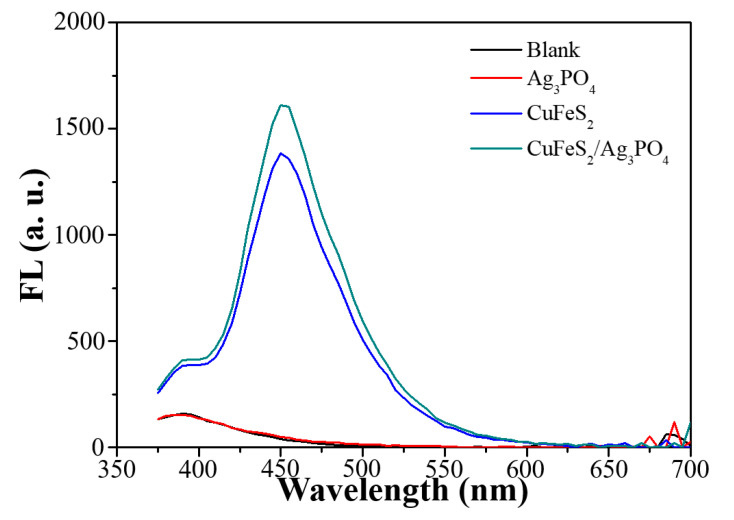
FL spectra of the different samples in the coumarin solution measured at λ_ex_ = 370 nm (each sample was illuminated for 10 min under visible light). Ag_3_PO_4_ (red), CuFeS_2_ (blue), and CuFeS_2_/Ag_3_PO_4_ composites (olive).

Hydroxyl radical production was further detected using the fluorescent luminescence (FL) technique to study the photo-Fenton reaction. The FL emission spectra, excited at 370 nm in the coumarin solution in the absence and presence of the prepared samples, were evaluated for 10 min of irradiation. Figure 15 shows that an FL signal was observed at 460 nm for each sample and that the maximum FL intensity was observed in CuFeS_2_/Ag_3_PO_4_. This suggests that CuFeS_2_/Ag_3_PO_4_ produced the highest amount of hydroxyl radicals among these three materials, thereby leading to more chemical reactions with coumarin to generate fluorescence [33]. Hence, the hydroxyl radical was considered the direct reactive oxidation species in the CuFeS_2_/Ag_3_PO_4_ composites for RhB degradation. Moreover, CuFeS_2_/Ag_3_PO_4_ composites with maximal degradation activity produced excess reactive hydroxyl radicals than CuFeS_2_, which is consistent with the previously discussed results.

**Scheme 1:**
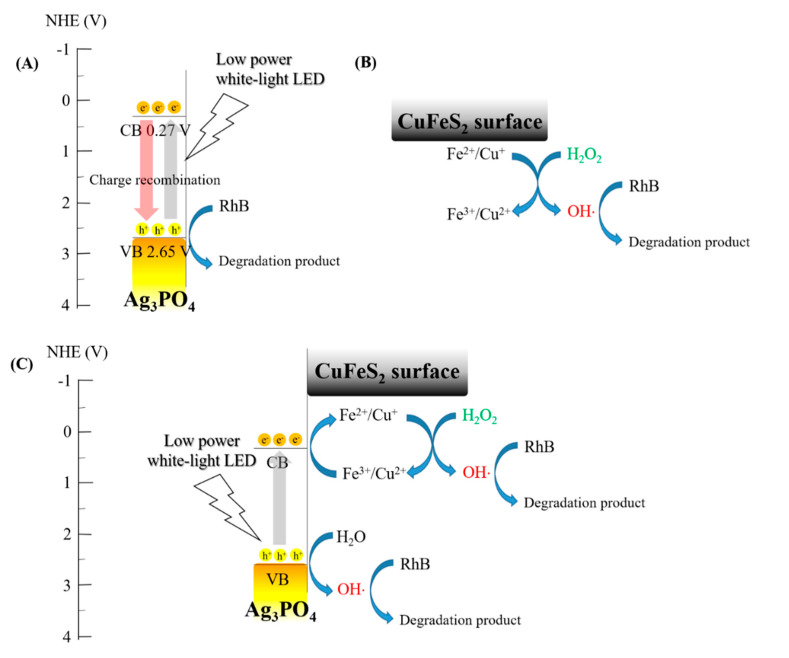
Transition of electrons and holes in the different degradation system of (**A**) Ag_3_PO_4_, (**B**) CuFeS_2_, and (**C**) CuFeS_2_/Ag_3_PO_4_.

On the basis of the results described above, the degradation mechanisms of the Ag_3_PO_4_, CuFeS_2,_ and CuFeS_2_/Ag_3_PO_4_ in the photo-Fenton reaction were proposed (Scheme 1). For Ag_3_PO_4_, the electron–hole pairs are generated under LED irradiation (Scheme 1A). The electrons could not form the oxygen radicals because of their higher position in the standard redox potential of oxygen/oxygen radical (0.13 V) than the CB potential (0.27 V). The photogenerated hole can produce the hydroxyl radical because the oxidation potential of the hydroxyl radical (1.99 V) is lower than the VB potential of Ag_3_PO_4_ (2.65 V). However, the recombination rate in Ag_3_PO_4_ is too fast to produce sufficient hydroxyl radicals by the holes. Thus, the holes are responsible for RhB degradation. The poor charge separation of CuFeS_2_ limits its ability to generate electron–hole pairs, but it is effective in generating hydroxyl radicals through the Fenton reaction (Scheme 1B). However, the Fenton reaction causes an increase in the oxidation state of Cu and Fe within the CuFeS_2_ solids, alters the crystal structure, and notably, weakens the integrity of the CuFeS_2_ solids. This is the main reason CuFeS_2_ is hardly reused in water treatment. When Ag_3_PO_4_ couples with CuFeS_2_ to form the CuFeS_2_/Ag_3_PO_4_ composites, the photogenerated electrons can be easily captured by the oxidized Fe^3+^/Cu^2+^ on the surface of CuFeS_2_, leading to the regeneration of Fe^2+^/Cu^+^ at the CuFeS_2_/Ag_3_PO_4_ interface (Scheme 1C). As a strong synergistic effect, the formation of hydroxyl radicals through the Fenton reaction on CuFeS_2_ and photogenerated hole on Ag_3_PO_4_ is favored to enhance the degradation activity and stability of CuFeS_2_/Ag_3_PO_4_ composites. In addition, we considered that the degradation mechanism for the prepared Fe_2_S_3_/Ag_3_PO_4_ and Cu_2_S/Ag_3_PO_4_ composites was similar to that of CuFeS_2_/Ag_3_PO_4_ composites. However, the degree of the charge separation efficiency, recombination rate of electron–hole pairs and the amount of the major reactive species were slight different, resulting in different degradation performance as shown in Figure 10.

### 3.5. Stability and Practical Applications of CuFeS_2_/Ag_3_PO_4_

**Figure 16:**
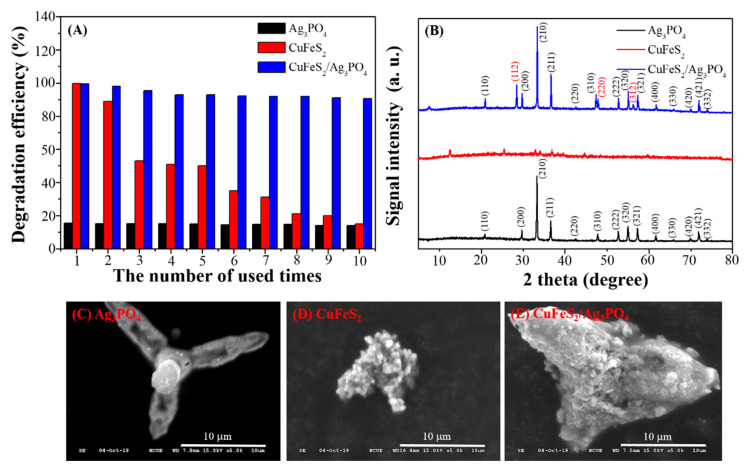
(**A**) Degradation efficiency under a 10 min reaction time for the recycle used test, (**B**) XRD patterns, and (**C**–**E**) the corresponding SEM images of the third used samples: Ag_3_PO_4_ (black), CuFeS_2_ (red), and CuFeS_2_/Ag_3_PO_4_ composites (blue).

The stability of the catalyst is an essential parameter for the development of practical water treatment applications. To investigate the stability of the prepared Ag_3_PO_4_, CuFeS_2_, and CuFeS_2_/Ag_3_PO_4_ composites, the results of cyclic RhB degradation tests were evaluated (shown in Figure 16A); in each cycle, RhB and H_2_O_2_ were reintroduced into the catalyst. In this study, RhB degradation by Ag_3_PO_4_ and CuFeS_2_/Ag_3_PO_4_ after ten cycles maintained a similar degradation efficiency (15.3% to 14% after ten cycles for Ag_3_PO_4_; 99.6% to 91.3% after ten cycles for CuFeS_2_/Ag_3_PO_4_), whereas that by CuFeS_2_ resulted in a considerable loss of efficiency (from 97.5% to 15.0% after ten cycles). Furthermore, the corresponding XRD results (Figure 16B) suggest a negligible change in the phase structure of Ag_3_PO_4_ and CuFeS_2_/Ag_3_PO_4_ samples after the repeated reactions, indicating the good stability of the samples. However, the initial phase structure of CuFeS_2_ disappeared after three reaction cycles. The SEM image shown in Figure 16D displays that the morphology of the CuFeS_2_ sample changed from an irregular sheet to spherical shape, indicating that the Fenton reaction caused structural and chemical change for CuFeS_2_ when it is used alone. Unlike CuFeS_2_, the comparison of SEM images for Ag_3_PO_4_ and CuFeS_2_/Ag_3_PO_4_ composites before and after reaction cycles only showed a slight change of morphology (Figure 16C,E). Evidently, the stability of CuFeS_2_ in the CuFeS_2_/Ag_3_PO_4_ composite improved considerably because of its coupling with Ag_3_PO_4_ nanoparticles as the photogenerated electrons in the CB of Ag_3_PO_4_ reduce Fe^3+^/Cu^2+^ ions and keep them intact in CuFeS_2_/Ag_3_PO_4_. This prevented the structural disintegration of CuFeS_2_ during the Fenton reaction, effectively demonstrating its reusability for catalysis.

**Figure 17:**
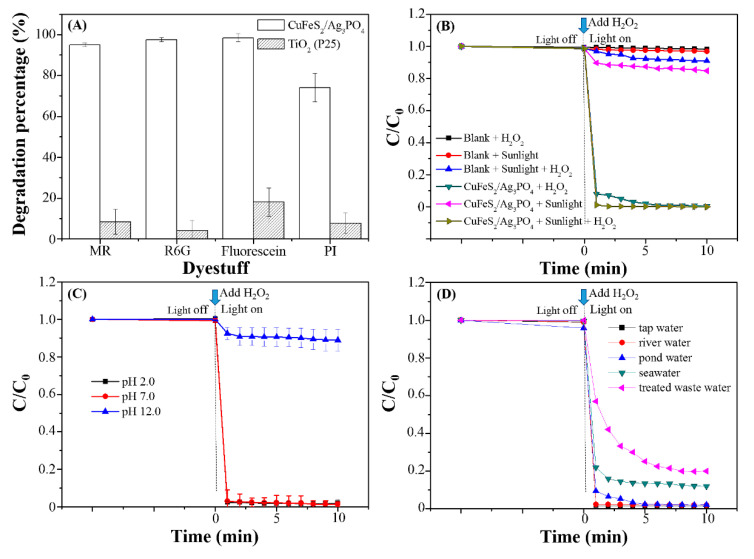
Photo-Fenton reaction of (**A**) different dyestuff by CuFeS_2_/Ag_3_PO_4_ composites and P25; (**B**) different degradation procedures in the absence and presence of CuFeS_2_/Ag_3_PO_4_ under sunlight irradiation; (**C**) different pH; and (**D**) different environmental water samples for RhB degradation by CuFeS_2_/Ag_3_PO_4_.

To assess the practical applications of CuFeS_2_/Ag_3_PO_4_, various organic dyes (MR, M6G, fluorescein, and PI) were degraded (Figure 17A). Compared with TiO_2_ (P25), CuFeS_2_/Ag_3_PO_4_ exhibited excellent degradation efficiency toward all selected dyestuffs, with nearly 95% degradation achieved within 10 min (except PI with only 78.5% degradation efficiency). In addition, the degradation performance of CuFeS_2_/Ag_3_PO_4_ under sunlight irradiation was evaluated from November to December 2019 from 11:00 a.m. to 2:00 p.m. daily at the National Changhua University of Education, Changhua, Taiwan. As shown in Figure 17B, sunlight-induced RhB degradation in the absence and presence of CuFeS_2_/Ag_3_PO_4_ was very poor without H_2_O_2_. However, using the sunlight-assisted Fenton reaction for RhB degradation achieved nearly 98.9% degradation efficiency within 1 min. This is because the combined UV and visible light in sunlight hastened the production of hydroxyl radicals in the presence of H_2_O_2_ and CuFeS_2_/Ag_3_PO_4_. With the promise of the sunlight-assisted Fenton reaction, the pH effect in the water samples on the degradation performance of CuFeS_2_/Ag_3_PO_4_ was also investigated. As shown in Figure 17C, the degradation efficiency decreased at pH 12.0 because the Fenton reaction was considerably hindered at a high pH as Fe^2+^ cations form inactive porphyrin ferryl complexes (FeO^2+^) in the alkaline solution [24]. Finally, the prepared CuFeS_2_/Ag_3_PO_4_ composites were used to degrade RhB in the environmental water samples (Figure 17D). CuFeS_2_/Ag_3_PO_4_ exhibited excellent degradation efficiency through the photo-Fenton reaction for RhB degradation, with nearly 90% degradation within 1 min. A notable difference in the degradation time for RhB was observed for the seawater and treated wastewater samples (90% and 80% degradation within 10 min, respectively) compared with the other environmental water samples (100% degradation within 10 min) probably because of the presence of anions or radical scavengers in the seawater and treated wastewater samples that reduced the degradation activity of CuFeS_2_/Ag_3_PO_4_. Nevertheless, the studies on the environmental water samples strongly support the benefits of this newly developed CuFeS_2_/Ag_3_PO_4_-based photo-Fenton water treatment option.

## 4. Conclusions

The currently prepared CuFeS_2_/Ag_3_PO_4_ composites exhibited higher RhB degradation efficiency through the photo-Fenton reaction than did Ag_3_PO_4_ and CuFeS_2_ alone. This high enhancement in the degradation efficiency was attributed to the synergistic effect in material stability and the hydroxyl radical production. The constituent Ag_3_PO_4_ in the newly developed composite not only provides the visible-light absorption ability in degrading organic compounds but also acts as a rich electron source to stabilize the crystal structure of CuFeS_2_ under light irradiation. Consequently, Cu^2+^/Fe^3+^ ions produced by the Fenton reaction can be reduced and regenerated into Cu^+^/Fe^2+^ ions, and the reactive hydroxyl radicals partially from the photogenerated holes of Ag_3_PO_4_ and predominantly from the Fenton reaction of CuFeS_2_ can be continuously produced to degrade organic compounds. The CuFeS_2_/Ag_3_PO_4_ composite has several attractive features not realized in the other reported photo-Fenton reactions (Table 2). First, the prepared CuFeS_2_/Ag_3_PO_4_ composite had 96% RhB degradation performance under low-power white LED illumination within 1 min. In addition, various dyestuffs (MR, R6G, fluorescein, and PI) with 95% degradation efficiency could be achieved. Through sunlight-assisted Fenton reaction, the RhB degradation efficiency was further improved to 98.9%. For the recycling used ability, the CuFeS_2_/Ag_2_PO_4_ composite is stable enough to be reused through the input of sustainable energy source. Hence, this study discovered the synergistic catalysis CuFeS_2_/Ag_3_PO_4_ and successfully demonstrates the application of the sunlight-assisted Fenton reaction on environmental water samples. The current findings can be used for the applications of advanced oxidation technology in wastewater treatment in the future.

## Figures and Tables

**Table 1:** Pseudo-first-order rate constants for rhodamine B (RhB) degradation by Ag_3_PO_4_, CuFeS_2_, and CuFeS_2_/Ag_3_PO_4_ composites at different conditions.

Series	Degradation Mode	Pseudo-First-Order Kinetic Equation	k(min^−1^)	R^2^
Ag_3_PO_4_	Photocatalytic reaction	y = 0.016x + 0.048	0.016	0.96
	Fenton reaction	y = 0.0026x + 0.0088	0.0026	0.92
	Photo-Fenton reaction	y = 0.0047x + 0.055	0.0047	0.80
CuFeS_2_	Photocatalytic reaction	y = 0.0007x − 0.039	0.0007	0.72
	Fenton reaction	y = 1.5x − 0.16	1.5	0.93
	Photo-Fenton reaction	y = 2.8x − 0.29	2.8	0.94
CuFeS_2_/Ag_3_PO_4_	Photocatalytic reaction	y = 0.018x − 0.0015	0.018	0.94
	Fenton reaction	y = 2.2x − 0.156	2.2	0.99
	Photo-Fenton reaction	y = 3.3x − 0.0322	3.3	0.97

**Table 2:** Comparison of the degradation performance and practical applications using the photo-Fenton reaction.

Samples	Preparation	Degradation Performance	Sunlight Irradiation	Target	Ref.
Citrate–CuFeS_2_	Microwave heating	90% degradation (0.2 g catalyst/50 ppm BPA) within 15 min (4 W fluorescent lamp)	-	BPA	[24]
FS–TiO_2_ disk	Dip-coating method	95% degradation (50 ppm phenazone (PNZ)) within 180 min (36 W UV light)	95% degradation of 50 ppm PNZ within 90 min	PNZ	[34]
Mined CuFeS_2_	Milling process	85% TOC conversion (1.0 g catalyst/0.5 mM tyrosol (TY)) within 60 min (10 W UV LED light)	-	TY	[19]
Fe–N–Ag–TiO_2_ clay bead	Surface impregnation method	-	77% degradation of 50 ppm cephalexin (CEX) within 60 min	CEX	[35]
FS/FA/TiO_2_ clay bead	Dip-coating method	89% degradation (50 ppm CEX) within 4 h (36 W UV light)	94% degradation (50 ppm CEX) within 3.5 h	CEX	[36]
Fe_2_O_3_–TiO_2_ film	Sol–gel method	80% degradation (10 ppm ciprofloxacin (CIPRO), sulfamethoxazole (SMX), and trimethoprim (TMP) mixture) within 240 min by a solar simulator (Solarbox Model 1500e)	-	CIPRO, SMX, TMP	[37]
CuFeS_2_/Ag_3_PO_4_	Cyclic microwave heating	96% degradation (20 mg catalyst/20 ppm RhB) within 1 min (2.5 W white-light LED)	99.8% degradation (0.15 g catalyst/15 ppm MB) within 6 min	RhB, MR, R6G, Fluorescein, PI, phenol	This study
